# Challenges faced by Arab women who are interested in becoming physicians

**DOI:** 10.1186/s13584-015-0029-4

**Published:** 2015-06-30

**Authors:** Bishara Bisharat, Abdalla Bowirrat

**Affiliations:** EMMS Nazareth Hospital, Faculty of medicine, Bar Ilan University-Galilee, P.O.Box: 11, Nazareth, 16100 Israel

**Keywords:** Arab physicians, Under-representation, Marginalization, Health system, Israel

## Abstract

Understanding the underlying reasons for the under-representation of Arab women within the health care system in Israel is crucial for creating future strategies for intervention, in order to minimize the gaps in the health care system and thus improve the medical services and health status.

Our commentary tries to shed light on the underrepresentation and the marginalization of the Arab women in society in general and in the medical field in specific.

## Commentary

### Background

The article by Keshet and colleagues [6] addresses the underrepresentation and the marginalization of Arab women in the medical field. It does so using an intersectionality approach which stresses the role of gender and ethnicity as a research paradigm to clarify the complexity of health inequities.

The manuscript discusses various underlying causes of the under-representation of Arab women in the health care workforce, such as tradition, employment opportunities and socioeconomic status. It examines the barriers that limit the involvement of Arab women in the medical field, based in part on the opinions of several Arab physicians and nurses who work in the field.

Keshet and her colleagues emphasize the importance of ethnic diversity within the health care workforce for decreasing health disparities, and for providing more professionals who are familiar with the language and culture of patients. This is important for improving communication, comfort level, trust and partnership among patients and practitioners, which in turn can lead to better use of appropriate health care and adherence to effective programs, resulting in improved health outcomes.

The term ‘under-represented in medicine’ refers to those racial and ethnic minority populations that are under-represented in the medical professions relative to their numbers in the general population [1].

Language and culture concordance improve the health care provided to the patients, patient-practitioner gender relations have been associated with improved patient’s health [2].

Dyads of patients-physicians of the same gender are characterized by a more encouraging communication style; both verbal (through positive statements and encouraging back channel responses) and nonverbal (nodding). Female physician/female patient dyads are also characterized by a calmer tone of voice, suggesting relative ease in interactions, and they produce the longest consultations, in which more talk occurs than in any other dyad combination [9].

This aspect of relationship is extremely important for females in traditional Arab society.

## The Arab minority in Israel

The Arab minority is the largest ethnic minority in Israel, comprising about 20 % of the population. In spite of being the largest ethnic minority in the country, the Arabs in Israel suffer from many inequalities compared to the counterpart Jewish majority.

Inequalities between Arab and Jewish citizens of Israel span all fields of public life and have persisted over time.

These include: lower socioeconomic status, higher unemployment rates, lower education levels and a long history of marginalization, disadvantage in income, education and employment [5, 7, 10].

In addition, the ongoing national conflict in the region and the inferior political status of the Arabs in Israel place tremendous pressures on this population [8].

## Reflections on the under-representation of Arab women in the health system

The article by Keshet and colleagues investigated the gender patterns of under-representation in medicine among Arab women, who are a minority within a minority. Accordingly, they suffer from double marginalization—being women within the Arab patriarchal society and belonging to an ethnic minority in the dominantly Jewish nation state of Israel [4]. Keshet and colleagues explored the underlying reasons for this under-representation in health care services, such as gendered education and employment patterns among the Arab minority in Israel.

Many theories have been suggested throughout the article in an attempt to understand the nature of this under-representation by trying to contribute in tackling this quandary.

We accept the notion that Arab physicians’ choice to follow medicine as a career is presented as an informed and practical choice. It constitutes a path to channel excellence among young Arabs whose exam scores are particularly high. Young Arabs are often sent to study abroad (among Israeli born Jewish physicians, 85 % studied medicine in Israel, compared to only 23 % among the Arab physicians) [3, 11].

The reason for this phenomenon is the relatively low percentage of Arab students accepted to study medicine in Israeli medical school due to restrictive prerequisites such as psychometric tests, difficult interviews in Hebrew language in addition to high marks in the matriculation exams. Therefore, Arab students who wish to pursue a medical care are often forced to face their fate and study abroad, away from their families. The Arab women tolerate double challenges: Beside the above-mentioned difficulties, they face the religious and traditional roles which prohibit them to be alone abroad and face the challenges of learning a new language, adapting to the new society’s customs and taking into consideration the economic challenge which, taken as a whole, limit them to study medicine abroad. Indeed, talented Arab male students are urged by their families to study and practice medicine abroad, also because the subject is considered prestigious and respectable in the community. Exceptional Arab student women, on the other hand, are channeled to teaching in their local communities.

Furthermore, men in the Arab society are viewed as the providers for their families while women on the other hand are responsible for taking care of the children, and to be at home, with the family. This makes it difficult for them to pursue medicine. The long studying years and the hard work conditions pose additional barriers.

This also brings us to the point that when a male Arab is not accepted to Israeli medical schools he will try to find an alternative abroad. Arab females don’t have such an option; therefore, they seek different fields of study in Israel, such as nursing or teaching. All this contributes to the under-representation of females in the medical field.

In nursing, there is no under-representation between Arabs and Jews (0.62 vs. 0.69 %). By comparison - in nursing, the absence of under-representation can also be explained by gender factors. Nursing is perceived as a feminine occupation and in Israel far more women (1.2 % of the population of women) than men (0.2 % of all men) are nurses. The rate of Arab female nurses (0.8 % of the population of Arab women) is lower than that of Jewish female nurses (1.2 %), but the gap is far smaller than among female physicians. Moreover, we find a higher rate of Arab (0.4 %) than Jewish (0.1 %) men who are nurses (*P* < .001). This small gap between Arab and Jews is attributed partially to the shortage of nurses in hospitals in general. This enhances the opportunities for Arab nurses to engage in this field. This phenomenon is also observed among Arab physicians today, in light of Israel’s current physician shortage.

We believe that socially driven custom, such as the family intervention against females’ choice to be engaged in medicine, must be resolved by increasing awareness, knowledge, and the understanding the importance of involving more females as professionals in the health care system. This action should be encouraged by the families and health policy makers. One of the suggested ways to increase the acceptance rate among the young Arab females is to expose them to preparation courses that may enhance their preparedness for the medical admission and for medical school as well.

## Conclusions

Nevertheless, we still hope that health policy makers will do tremendous efforts to close the gaps and decrease the inequalities between Arab and Jewish citizens of Israel especially in the field of medicine by giving more opportunities. It will be important to track changes over time in the rates of acceptance, by gender, of both Arabs and Jews to Israeli medical schools. As part of that broader analysis, it will be particularly interesting to see whether the recent establishment of the new medical school in the Galilee has a significant impact on these patterns and trends. It will be fascinating to observe increasing rates of Arab female acceptance in the new medical school in the Galilee and to see whether this rate become equal to the rate for their Jewish counterparts.

Notwithstanding the fact that Arab representation in almost all fields in Israel was very low, an increasing engagement is noticed in the Arab society among both gender in the scientific fields, especially in medicine. This new wave of change is due to the transformation and modernization that Arab society is undergoing, in spite of all the obstacles. In fact, nowadays more and more Arab women are engaging in the field of medicine both in Israel and abroad.
